# Utility of Polygenic Risk Scores (PRSs) in Predicting Pancreatic Cancer: A Systematic Review and Meta-Analysis of Common-Variant and Mixed Scores with Insights into Rare Variant Analysis

**DOI:** 10.3390/cancers17020241

**Published:** 2025-01-13

**Authors:** Georgios Ioannis Verras, Zaed Z. Hamady, Andrew Collins, William Tapper

**Affiliations:** 1 School of Human Development and Health, Faculty of Medicine, University of Southampton, Southampton SO16 6YD, UK; z.hamady@soton.ac.uk (Z.Z.H.); a.r.collins@soton.ac.uk (A.C.); 2 Department of General Surgery, University Hospital Southampton, Southampton SO16 6YD, UK

**Keywords:** pancreatic cancer, polygenic risk scores, genetic scores, clinical risk scores, risk stratification, pancreatic adenocarcinoma, risk prediction

## Abstract

Introduction: Pancreatic cancer remains among the top five causes of cancer-related mortality. Only 18% to 20% of patients present with early-stage, potentially curable disease. Patient risk stratification is critical to increase the number of individuals eligible for surgery. Polygenic risk scores (PRSs), combined with clinical risk factors, offer a promising approach to assess the lifetime risk of pancreatic cancer. This systematic review synthesizes the results of all published PRSs and mixed models for pancreatic cancer. Methods: We systematically searched the MEDLINE, Embase, Ovid and Web of Science databases. The odds ratios reported between risk quintiles were transformed to an OR per SD increase in risk score. Reported AUCs were synthesized and compared between the PRSs and mixed models. Results: Twenty-seven studies were identified for formal synthesis. The PRSs yielded an OR of 1.40 (1.28–1.53) for pancreatic cancer, while mixed models incorporating clinical risk factors showed a higher OR of 1.58 (1.34–1.88). The AUC for the PRSs was 0.61 (0.58–0.65), compared to 0.70 (0.61–0.80) for the mixed models. The predictive power of PRS models was positively correlated with the number of SNPs included in the studies. Ancestry significantly influenced prediction accuracy, with an OR of 1.42 (1.30–1.56) in Caucasian populations compared to 1.21 (0.77–1.90) in Asian populations. Conclusions: Adding clinical risk factors to PRS models significantly increases their predictive capability. Further research is needed to identify a comprehensive set of risk SNPs, enhance accuracy across diverse populations and incorporate rare genetic variants. These advancements have the potential to significantly boost the predictive accuracy of PRSs and mixed models.

## Simple Summary

Pancreatic cancer remains one of the highest-mortality cancers worldwide, with most patients diagnosed at an advanced stage, making curative surgical resection impossible for many. As research efforts focus on early detection, we reviewed the current literature on the use of genetic risk markers and patient risk factors to estimate a person’s lifetime risk via polygenic risk scores (PRSs) and mixed models that combine PRSs with clinical risk factors. Our analysis demonstrates that combining genetic data with patient risk factors significantly improves risk prediction. Through a systematic review and meta-analysis, we show that predictive models perform better in populations with Caucasian ancestry compared to non-Caucasian ancestry. Additionally, increasing the number of genomic markers improves the predictive power of these models. Finally, we consider the potential of rare variants to further enhance the accuracy of these risk prediction models for pancreatic cancer.

## 1. Introduction

Pancreatic adenocarcinoma is the most common histological subtype of pancreatic cancer, representing approximately 85% of all cases [1,2,3]. Despite advancements in treatment modalities, including advanced chemotherapy regimens and novel drug delivery approaches [4], surgical management remains the only potentially curative option [1,2,3]. However, most patients present with a late-stage disease, which is often inoperable due to the insidious and asymptomatic progression of the disease [2,3,4,5]. Efforts to improve early disease detection through biomarkers and advanced imaging are ongoing, but significant improvements in diagnostic rates have yet to be achieved. Early diagnosis is key as this would constitute the disease operable, and thus potentially curable.

Environmental risk factors like tobacco use, obesity, diabetes mellitus, chronic pancreatitis and alcohol consumption are all well-established risk factors for the development of pancreatic cancer [5]. Additionally, pancreatic adenocarcinoma is driven by genetic and epigenetic changes that promote carcinogenesis [5]. Up to 10% of patients report an affected first-degree relative, indicating that there is a moderate component of genetic variation in its risk of development [5].

### 1.1. Risk Stratification and Early Detection

Improving early diagnosis hinges on effective risk stratification, which could lead to the development of targeted screening programs. Several research groups have explored the development of polygenic risk scores (PRSs) to predict the risk of pancreatic cancer [6,7,8,9,10,11,12,13,14]. PRSs estimate an individual’s genetic risk of disease based on the combined effect of single nucleotide polymorphisms (SNPs) identified through genome-wide association studies (GWASs). While individual SNPs usually have weak associations with disease, their additive effect helps assess pancreatic cancer heritability and could be used to stratify patients into different risk prediction categories. Large case–control studies have developed PRS models with varying predictive capabilities and performance. Enhancing these scores by incorporating known clinical risk factors has further increased their predictive performance, especially in specific patient subsets such as patients with diabetes [6,7].

### 1.2. Expanding the Model with Rare Variants

In addition to common variants, rare genetic variants also play a role in the heritability and progression of pancreatic cancer [8,9,10,11,12]. Incorporating rare variants into polygenic scores has been explored in other diseases such as breast cancer, colorectal cancer and type 2 diabetes [13,14]. Studies have also suggested that individuals with low polygenic risk based on common variants are more likely to harbor rare, high-risk variants [14]. As a result, common-variant PRS models could be used to select patients for next-generation sequencing to identify these rare, disease-associated variants.

### 1.3. Objective

This systematic review and this meta-analysis aim to synthesize the current literature regarding PRSs and pancreatic adenocarcinoma. The primary goal is to assess the association between common-variant PRSs and pancreatic cancer risk using a meta-analysis of their reported odds ratio (OR) or hazard ratio (HR), as well as their corresponding 95% confidence intervals. Additionally, we will evaluate the role of rare variants (MAF < 0.01) as predictors of pancreatic adenocarcinoma risk.

## 2. Materials and Methods

This systematic review was conducted in accordance with the Preferred Reporting Items for Systematic Reviews and Meta-Analyses (PRISMA) guidelines. The systematic review protocol for common and rare variants PRS, the formal synthesis of the review results and the meta-analysis were all registered on PROSPERO (ID: CRD42024576795).

### 2.1. Search Strategy

All relevant studies were identified through database searches, as well as a citation screening. The systematic review comprised two different searches for an easier interpretation and discussion of the findings. Firstly, we conducted a systematic search on all studies that had built and assessed a common-variant PRS on pancreatic adenocarcinoma, with or without the inclusion of clinical risk factors (mixed scores). Secondly, a separate search was conducted on studies investigating the association of rare variants (MAF < 0.01) with pancreatic adenocarcinoma. Due to the lack of studies reporting rare variants as part of the PRSs for pancreatic adenocarcinoma, studies investigating rare variant associations were considered separately. For the purpose of both searches, the MEDLINE, Embase, Ovid and Web of Science databases were used. The full search queries for both research questions are presented in  Supplementary Tables S1 and S2, respectively. The searches included articles published up to 3 June 2024.

### 2.2. Study Selection

For the purposes of this review, the initial study search and screening were conducted by GIV at the title, abstract and full text stages. The further assessment of the included studies at the full text stage was conducted by ZH, WT and AC. Disagreements regarding the evaluation of the studies’ quality and relevance were resolved by consensus. The inclusion criteria for both literature searches included the following: (1) adult participants aged 18 or older, (2) pancreatic cancer or adenocarcinoma specifically reported as endpoint outcome and (3) studies published in English. Studies were excluded if (1) they were of poor design as viewed through the bias and study quality assessment process or (2) were systematic reviews, editorials, opinion articles, letters to the editor or conference papers.

For the first search process regarding PRSs with common variants, studies were also excluded if (1) they reported on monogenic associations, (2) they calculated the PRS from less than 2 loci or (3) they had a small sample size (defined as <100 individuals). For the systematic review of rare variants in pancreatic cancer, studies were additionally excluded if (1) they included SNPs of MAF > 0.01 (as reported in dbSNP) or (2) reported on benign or likely benign variants.

### 2.3. Quality and Risk of Bias Assessment

The risk of bias of each study was assessed using the Prediction Model Risk Of Bias Assessment Tool (PROBAST). The methodological quality of all studies was also assessed by utilizing the Critical Appraisal Skills Programme (CASP) checklists for methodological rigor. Disagreements between reviewers were resolved by consensus.

### 2.4. Target Outcomes and Data Extraction

For the purposes of this literature review, we included all studies fitting the above-mentioned criteria, looking into PRSs for pancreatic cancer, as well as rare variants associated with the risk of developing pancreatic cancer. The outcome (phenotype) of interest in both cases was the development of a pancreatic cancer of any subtype (excluding neuroendocrine tumors), with pancreatic adenocarcinoma being the most investigated. To facilitate data extraction, a specific form was constructed using Microsoft Excel that included the first author name and year of publication, the patient cohort or biobank that each study was based on, the clinical risk factors included in the mixed scores (in studies investigating PRS + clinical risk factors), the number of SNPs used to construct each PRS, the number of included individuals, the presence of a replication/validation phase following the construction of the PRS and finally the reported risk estimates for each score, including the 95% confidence intervals. We also extracted tables of information on a “per risk strata” basis from each study, including the number of cases and controls included in the development of each risk score, the risk level and the reported risk estimates.

To complete our systematic review on rare variants associated with pancreatic adenocarcinoma, we extracted information on the genes and rare variants identified by each study. The primary focus was to evaluate the reported effect sizes of these rare variants and the development of pancreatic cancer. However, quantitative synthesis in the form of a meta-analysis was not feasible, as combining summary metrics from associations involving variants in different genes would be difficult to interpret.

### 2.5. Statistical Analysis

The meta-analysis focused on studies reporting on common-variant PRSs for pancreatic cancer. We performed a random effects meta-analysis using the DerSimonian and Laird inverse variance method to account for variability across studies [11]. Heterogeneity was measured using Higgins’s I statistic. Where risk estimates were presented as estimates between two categories of PRS-based risk stratification, they were converted to risk per standard deviation using the Greenland and Longnecker approximation [15], a method previously used in PRS meta-analysis. This conversion facilitated synthesis and improved the interpretability of the standardized odds ratios. For each study, the conversion to per-SD risk was undertaken using the doresmeta package in R version 4.4.1 and the MAJOR add-on in Jamovi version 2.3.28. Where reported, the AUC of each risk score was also included in a separate pooling synthesis and compared between the PRSs and mixed scores.

To examine the impact of adding clinical risk factors to PRSs, creating mixed scores, we performed a subset meta-analysis comparing studies that used PRSs alone versus those incorporating clinical risk factors. Chi-squared tests were used to compare the pooled results by model type or ancestry.

Between-study heterogeneity was further explored by identifying outlying studies contributing significantly to the variance. The find.outliers function in the dmetar R package was used to detect studies with 95% CIs lying outside the pooled 95% CI. Outliers were automatically removed to improve consistency. We also assessed heterogeneity based on publication year, population ancestry and the number of SNPs utilized in each study. Publication bias was visualized and assessed using funnel plots, as well as graded funnel plots.

Sensitivity analyses were conducted on patient subsets by ethnicity to assess differences in model performances between ancestries.

Finally, the effect of the publication year and number of SNPs on the reported effect sizes was also assessed by employing meta-regression techniques to the previously mentioned meta-analyses. A linear regression model was fitted, with the number of SNPs or publication year as predictors and the studies’ effect size as the outcome. Meta-regression allows an estimation of the effect of the number of SNPs and the year of publication as regression coefficients, as well as the degree of observed heterogeneity attributed to these variables. All analyses were performed using the R language, and the packages meta version 8.0, dosresmeta version 2.0.1, dmetar version 0.1.0, ggrepel version 0.9.6 and metafor version 4.6 were utilized.

## 3. Results

### 3.1. Common-Variant Polygenic and Mixed Risk Scores on Pancreatic Cancer

**Figure 1:**
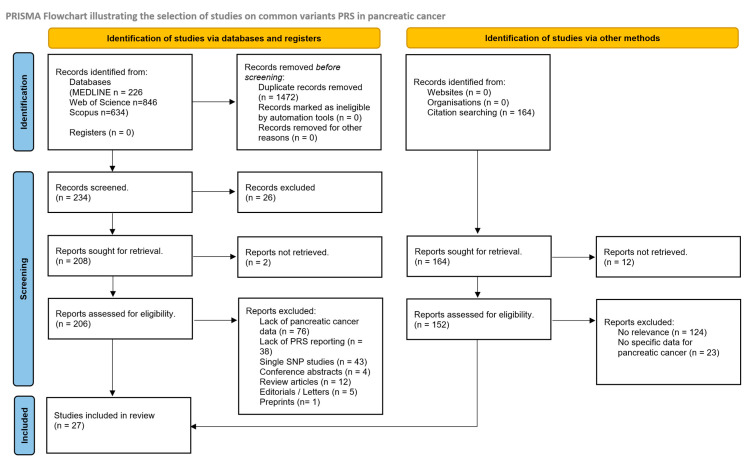
PRISMA flowchart for common-variant PRSs in pancreatic cancer. Taken from Page MJ, Mc Kenzie JE et al. [16].

In total, 27 studies were included in this systematic review regarding common-variant PRS studies in pancreatic cancer. Initially, a total of 386 articles were retrieved following the database search, deduplication and citation screening (234 from databases and registers + 152 from other methods). Out of these, 213 were assessed for inclusion, following title screening. The PRISMA flowchart for this literature search can be seen in Figure 1.

Following a removal of duplicates from the three databases, 1472 duplicate records were deleted. From the initial literature search, a total of 76 studies were removed on the basis of a lack of pancreatic cancer data references, 38 studies failed to report full PRSs, 43 studies reported only single SNP associations and another 22 studies were of an inappropriate study type for their inclusion in this review. An additional 152 studies were retrieved from citation searching and assessed for inclusion in this study, with 5 studies being included in the final synthesis. The included studies in the systematic review regarding common-variant polygenic and mixed scores in pancreatic cancer can be seen in Table 1.

### 3.2. Rare Variant Association Studies on Pancreatic Cancer

**Figure 2:**
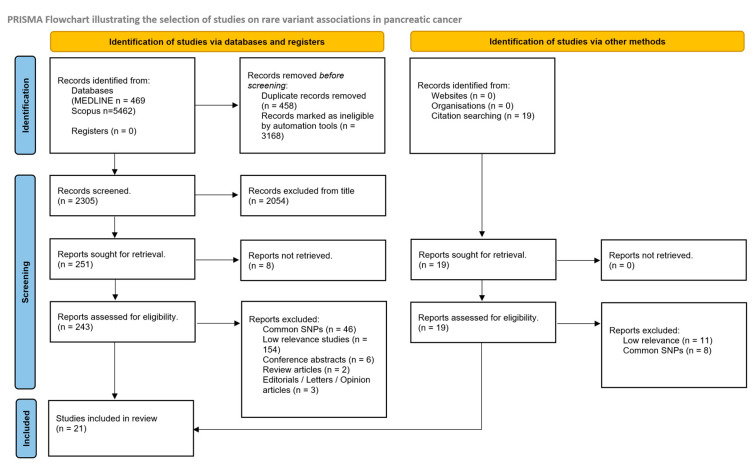
PRISMA flowchart for rare variant association analysis in pancreatic cancer. Taken from Page MJ, Mc Kenzie JE et al. [16].

For the systematic review of rare variants and pancreatic adenocarcinoma, a total of 21 studies were included in the final list of articles. Initially, a total of 251 studies were assessed following title exclusion, and 19 more were retrieved through citation searching (Figure 2). Forty-six studies were excluded due to the investigation of common rather than rare SNPs.

Following a full-text assessment, 154 studies were excluded due to low relevance (i.e., not focused on pancreatic cancer, cases studies involving single patients or investigations of rare molecular and histopathological subtypes of pancreatic cancer). Lastly, five more articles were excluded due to being of an inappropriate type. The remaining studies examining rare variants in relation to pancreatic cancer are listed in Table 2.

### 3.3. Meta-Analysis of Common-Variant PRS and Mixed Scores

**Figure 3:**
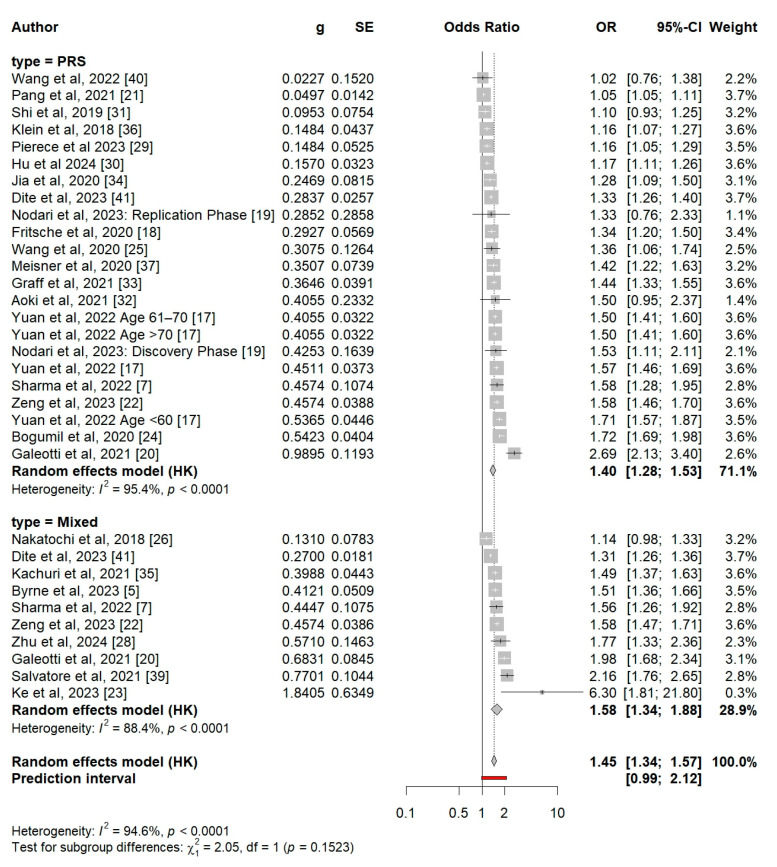
Synthesis of polygenic and mixed scores’ odds ratios (ORs) and 95% CIs. Legend: The size of the rectangle corresponds to the study weight, and the horizontal lines indicate the confidence intervals. Light grey text indicates group metrics. Bold text indicates overall synthesis metrics. Red line indicates prediction interval of calculated ideal model.

In total, 23 PRS-only models and 10 mixed models were included. Figure 3 presents a comparative synthesis of the standardized ORs from these models. The odds ratios per SD ranged from 1.02 to 2.69, with a small study by Ke et al. reporting an OR of 6.3. The pooled OR per SD increase in the PRS-only models was 1.40 (95% CI 1.28–1.53), while for the mixed scores it was 1.58 (95% CI 1.34–1.88). Although there was a modest increase in the average OR when clinical risk factors were incorporated, the comparison between the two groups did not reach statistical significance (*p* = 0.15), and both subgroups showed considerable heterogeneity (Figure 3). The overall pooled OR across the two study groups was 1.45 (95% CI 1.34–1.57).

**Figure:**
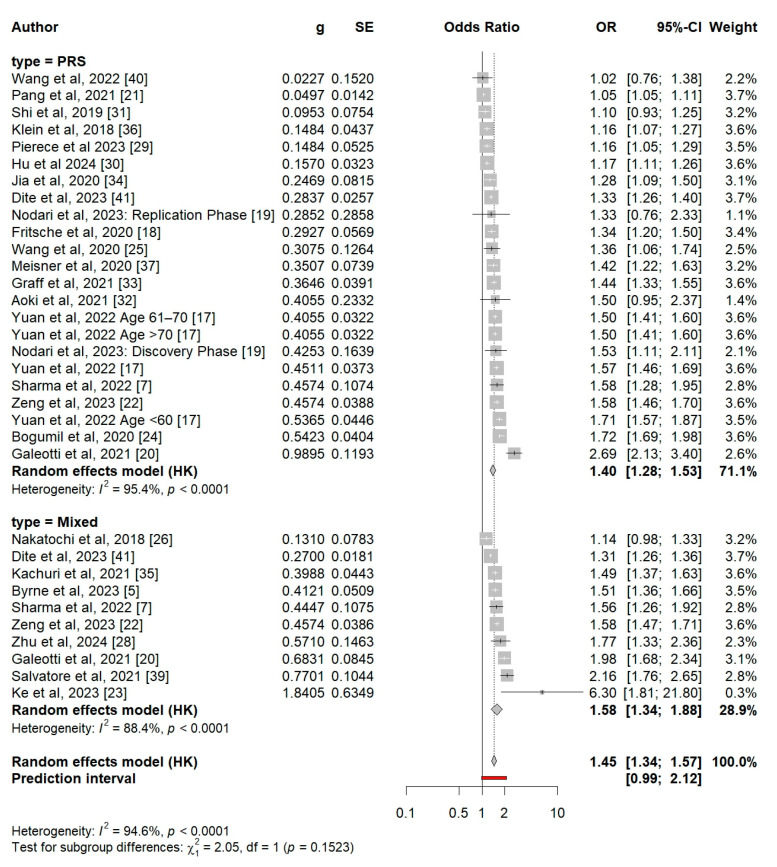


### 3.4. Meta-Analysis of AUCs from Common-Variant PRSs

**Figure 4:**
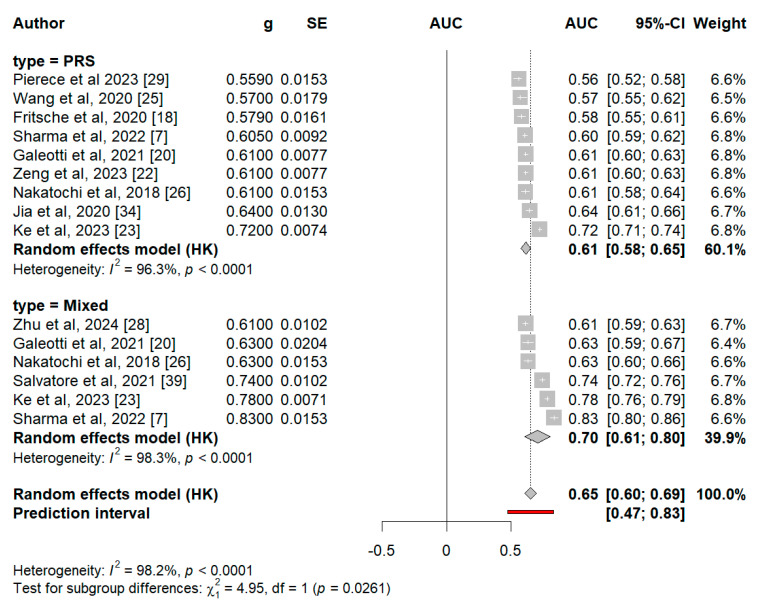
Pooled AUC scores by model type. Legend: The size of the rectangle corresponds to study weight, and horizontal lines indicate the confidence intervals. Red line indicates prediction interval of calculated ideal model.

AUC values were reported in nine PRS-only studies and six mixed score studies. A comparative meta-analysis yielded a pooled AUC of 0.61 (95% CI 0.58–0.65) for the PRS-only models and 0.70 (95% CI 0.61–0.80) for the mixed models (Figure 4). The inclusion of clinical risk factors led to a statistically significant improvement in predictive performance (*p* = 0.03). The overall pooled AUC across both the PRS-only and mixed models was 0.65 (95% CI 0.60–0.69), highlighting the benefit of combining genetic and clinical data for more accurate risk stratification.

**Figure:**
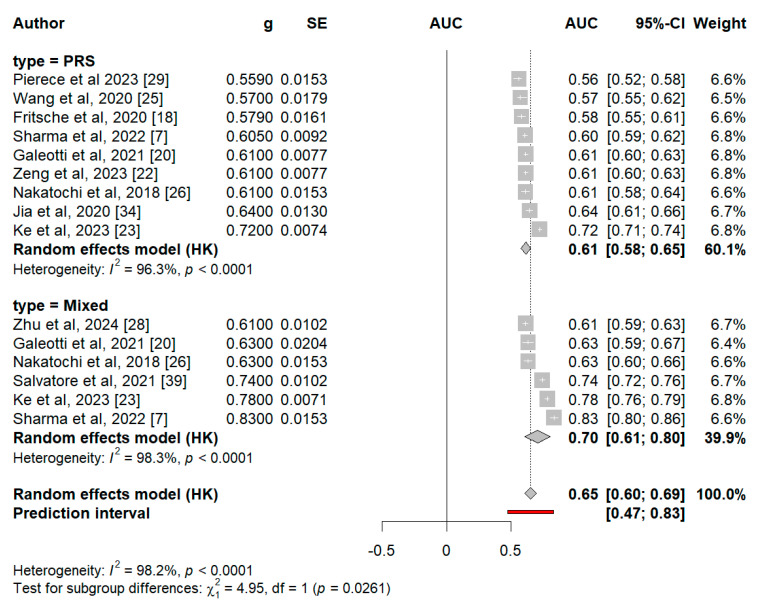


### 3.5. The Effect of Ancestry on Risk Score Performance

**Figure 5:**
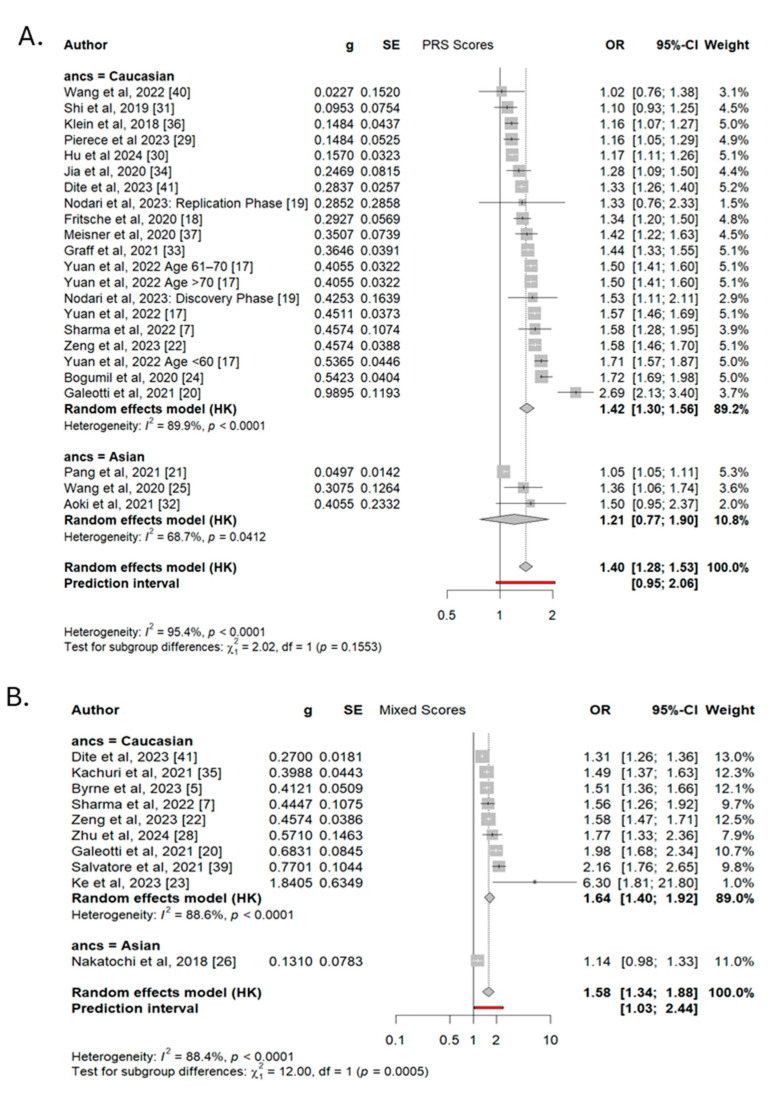
Comparison of pooled PRS estimates (**A**) and mixed scores (**B**) between Caucasian and Asian populations. Legend: The size of the rectangle corresponds to study weight, and horizontal lines indicate the confidence intervals. Red line indicates prediction interval of calculated ideal model.

Most of the included studies developed polygenic and mixed risk scores using data from populations of predominantly Caucasian/European ancestry. Only three PRS models were developed based on individuals of Asian ancestry (Pang et al. [20], Wang et al. [24], Aoki et al. [31]). A post hoc subgroup analysis comparing the scores between ancestry groups is shown in Figure 5A.

The pooled OR per SD increase in PRSs for studies of Caucasian ancestry was 1.42 (95% CI 1.30–1.56), compared to 1.21 (95% CI 0.77–1.90) for Asian-origin populations. Despite the observed OR difference of 0.21 between the ancestry subgroups, the statistical comparison was not significant (*p* = 0.16). Both subgroups, as well as the pooled estimate, exhibited high levels of heterogeneity (Figure 5).

Studies that combined PRSs with clinical risk factors had limited representation from Asian populations, with only one study by Nakatochi et al. [25] developing a mixed score in this group. The comparison between ancestry subgroups in the mixed models revealed a statistically significant difference between the ORs of 1.64 (95% CI 1.40–1.92) and 1.14 (95% CI 0.98–1.33) for the Caucasian and Asian populations, respectively. However, this difference in effect size should be interpreted cautiously, as the Asian subgroup consists of a single study. In addition, the adjusted OR per SD increase for Nakatochi et al. [25] included the null value within its 95% CI, further reducing the reliability of the effect size estimate (Figure 5B).

**Figure:**
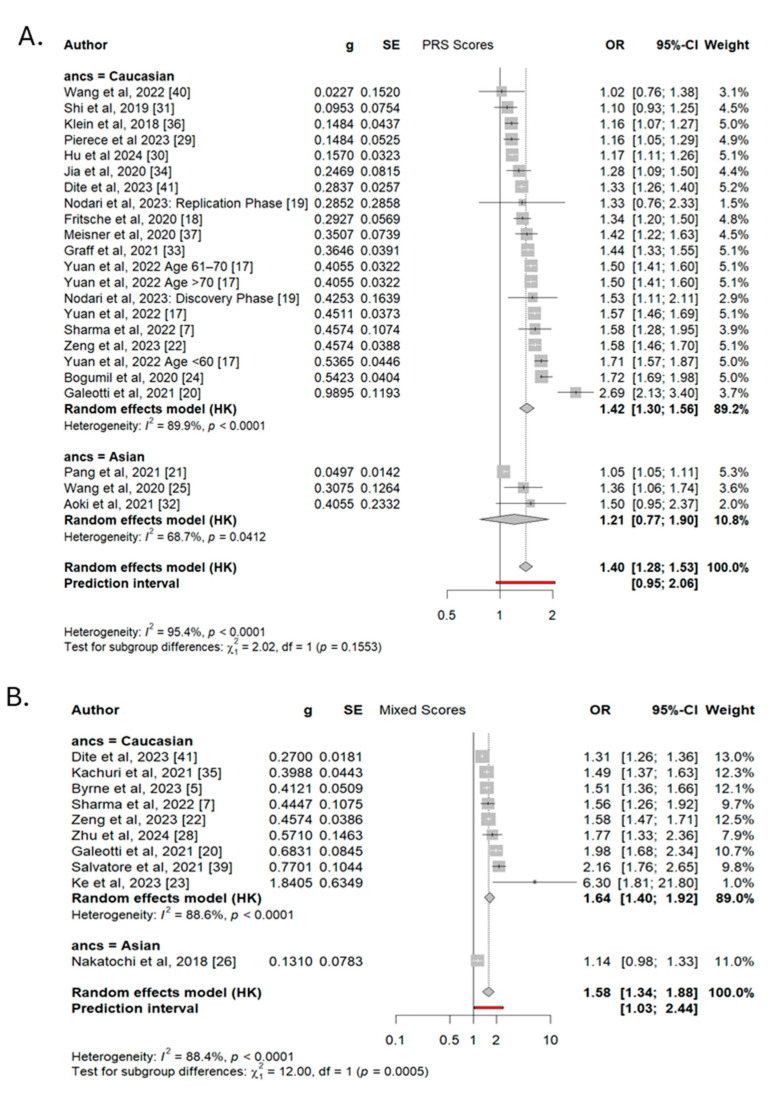


The AUC metrics by ancestry exhibited a similar difference as reported for the standardized OR increase in the PRS and mixed scores. Models developed from Caucasian ancestry populations were found to have higher pooled AUC estimates in the prediction of pancreatic cancer occurrence; the PRSs achieved a pooled AUC of 0.62 (95% CI 0.57–0.67), with mixed scores exhibiting a calculated AUC of 0.72 (95% CI 0.60–0.84) (Supplementary Figures S1 and S2). In contrast, models developed in populations of Asian ancestry achieved inferior predictive scores, with an AUC of 0.59 (95% CI 0.34–0.84) and 0.63 (95% CI 0.60–0.66) for the PRSs and mixed scores, respectively. The previously exhibited trend in the sub-optimal prediction metrics for pancreatic cancer was repeated in the AUC metrics.

### 3.6. Exploring the Effect of SNP Number and Publication Year in Model Performances Through Meta-Regression

**Figure 6:**
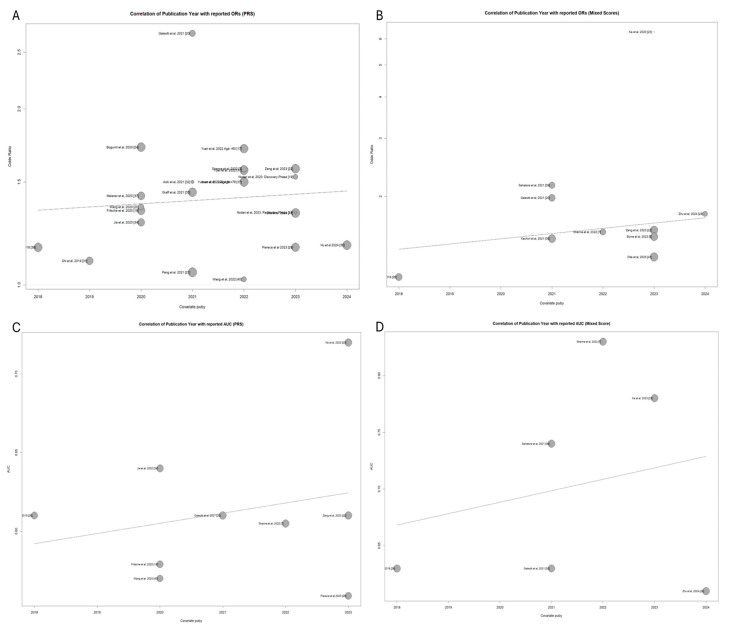
Effect of publication year on estimated effect sizes for standardized ORs in PRS studies (**A**), standardized ORs in mixed score studies (**B**), AUC in PRS studies (**C**), AUC in mixed score studies (**D**).

The next phase of the post hoc analysis involves conducting a meta-regression to examine the relationship between publication year and the reported effect size for both common-variant PRS and mixed models. This method allows an assessment of how much of the heterogeneity within the meta-analysis can be attributed to publication year. Figure 6 presents bubble plots displaying the study effect sizes (standardized OR) and AUC over the publication year. There is a positive correlation between publication year and effect size in most of the associations, as shown by the moderately positive slope of the trend lines.

**Figure:**
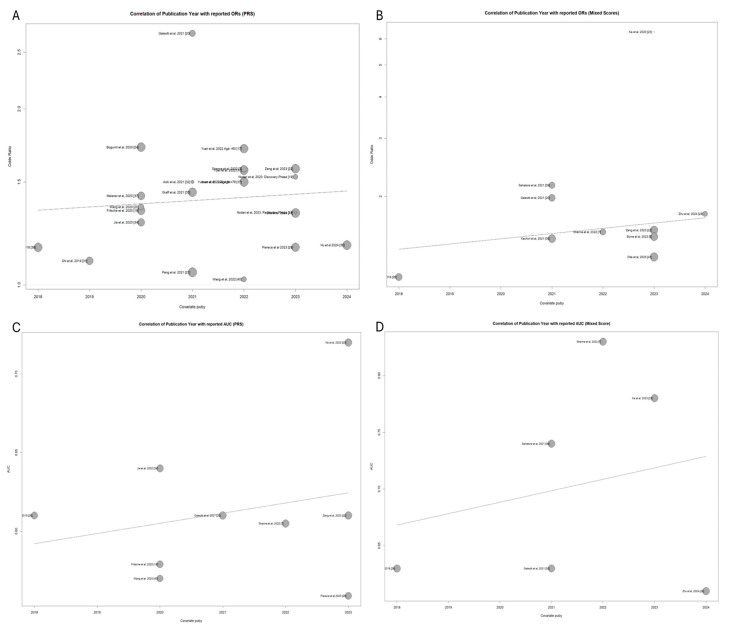


There is a moderate positive correlation between the publication year and reported PRS effect size; however, it does not constitute a significant predictor for this analysis (*p* = 0.63). The PRS study by Galeotti et al. [19] is identified as an outlier.

**Figure 7:**
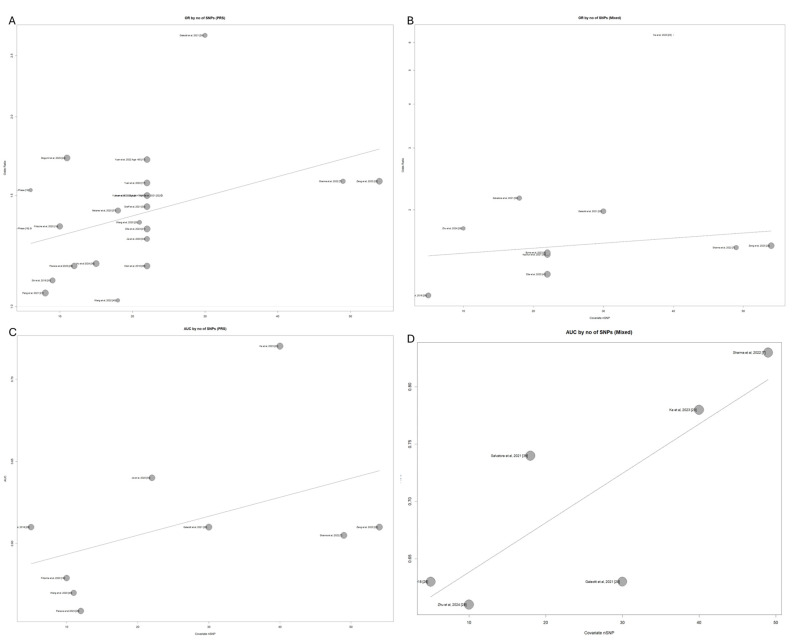
Correlation of number of SNPs included and reported study effect size in PRS-only (**A**) and mixed model studies (**B**). Correlation of number of SNPs utilized to build risk scores and reported AUC metrics in PRS-only (**C**) and mixed score studies (**D**). Legend: Studies by Sharma et al. [6] and Zeng et al. [21] are identified as outliers, achieving the highest number of SNPs included.

A similar association can be seen when plotting effect sizes by publication year for the mixed models (Figure 7B). There is a positive association between publication year and the reported standardized OR increase; however, publication year is not a significant predictor of effect size estimates (*p* = 0.46).

**Figure:**
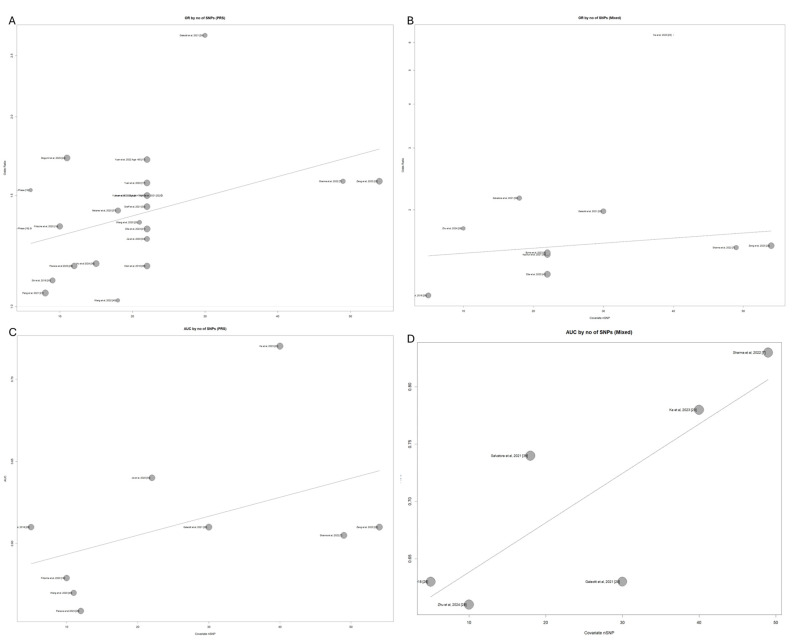


The mixed model study by Ke et al. [22] is identified as an outlier of the high reported ORs, although being of small overall weight in the pooled analysis. Following the regression analysis for publication year as a meta-analysis covariate, there was no heterogeneity attributable to publication year for any of the meta-analyses.

The number of SNPs included in the polygenic risk scores is an important potential covariate of increased predictive capability. This was assessed using meta-regression modeling to fit a model utilizing the number of SNPs for each model as a univariable continuous predictor of the reported metrics (OR or AUC).

The number of SNPs included in the PRS-only models shows a consistent positive association with the increased standardized ORs across the studies (Figure 7). The meta-regression analysis confirms that the number of SNPs is a significant predictor of effect size in the PRS-only models (*p* = 0.043), indicating that incorporating more SNPs tends to improve the predictive power of these scores. However, SNP number was not associated with the effect size of the mixed models (*p* = 0.408). This suggests that in mixed models, the addition of clinical factors may dilute the impact of the SNP count on predictive performance.

This analysis also reveals that a proportion of the heterogeneity observed between the PRS studies can be attributed to the number of SNPs included in each model. It was estimated that 18.29% of the heterogeneity previously identified (see Figure 3) can be explained by the number of SNPs used in each study. This indicates that SNP quantity is an important factor contributing to the variability in effect sizes reported across different PRS studies.

Conversely, when looking at the effect of SNP number in mixed models, they were not a significant predictor of the standardized increase in OR for pancreatic cancer (*p* = 0.54). The association between the reported OR and the number of SNPs in mixed models can be seen in Figure 7B.

The same trends can be seen when we look at the association between the number of SNPs and reported AUCs from the PRS-only and mixed score studies (Figure 7).

The meta-regression analysis for the AUCs reported by polygenic and mixed risk scores did not reveal a significant association between the number of SNPs used and prediction metrics (*p* = 0.2368 and 0.0613, respectively). A moderate percentage of between-study heterogeneity, however, could be attributed to the number of SNPs for both study subgroups (59.2% for the AUC analysis).

### 3.7. Addressing Heterogeneity Through Study Outlier Identification

**Figure 8:**
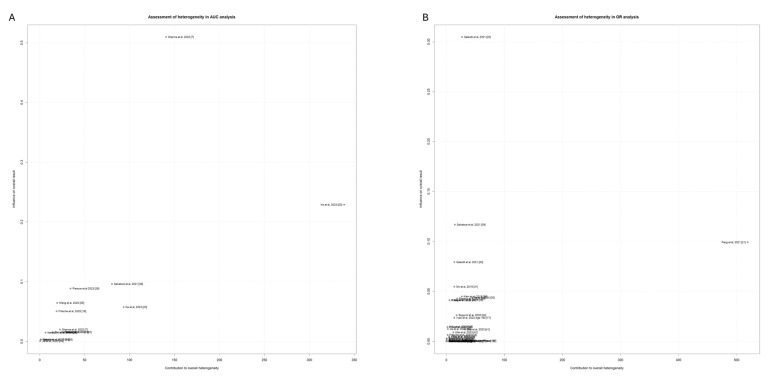
Baujat plot of studies utilized in OR analysis (**A**). Baujat plot of studies utilized in AUC analysis (**B**).

To further narrow down the source of between-study heterogeneity, we conducted a sensitivity analysis to identify outlying studies within the two meta-analyses that contributed a high degree of heterogeneity. A diagnostic plot of the study’s contribution to the Q-statistic for heterogeneity versus the influence of each study was used to visualize individual contribution to overall heterogeneity (Baujat plot) (Figure 8).

**Figure:**
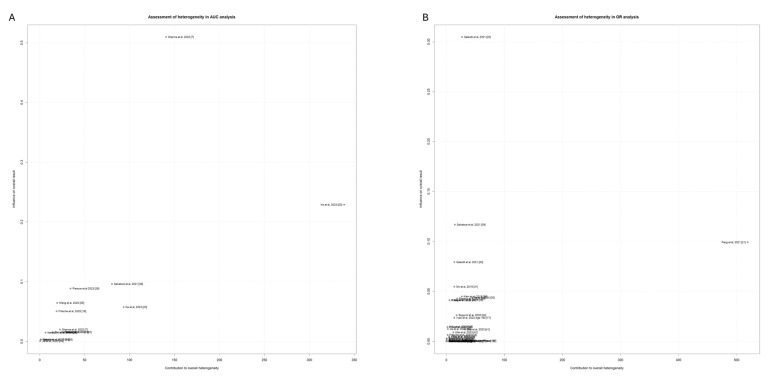


There are some studies that stand out after plotting individual heterogeneity contributions. The study by Pang et al. [20] appears as an outlier of high heterogeneity in the OR meta-analysis. Galeotti et al. [19] is shown to have the highest attributable influence on the pooled results while maintaining a low contribution to heterogeneity. For the AUC meta-analysis, the study by Ke et al. [22] is identified as an outlying contributor to the total heterogeneity of the analysis, while Sharma et al. [6] achieves the highest contribution to the pooled estimates, with a moderate contribution to overall heterogeneity.

The identification and removal of outlying studies of high heterogeneity was performed to assess the degree of heterogeneity individual studies could account for (see Section 2.5). For the OR meta-analysis between the PRS-only and mixed studies, the studies by Galeotti et al. [19], Ke et al. [22], Nakatochi et al. [25], Shi et al. [30] Klein et al. [35], Yuan et al., 2022 [16], Pang et al., 2021 [20], Bogumil et al. [23], Pierece et al. [28], Hu et al. [29] and Salvatore et al. [38] were identified as influential outliers and were removed from the analysis. The resulting new heterogeneity following this exclusion was 71.2% overall. The comparative results of the pooled effects following this influential study removal can be seen in Supplementary Table S3.

Similarly, for the AUC meta-analysis, the studies by Sharma et al. [6], Ke et al. [22], Pierce et al. [28] and Salvatore et al. [38] were identified as influential to heterogeneity and were removed to improve heterogeneity. Heterogeneity was reduced from 98% to 47% following exclusion ( Supplementary Table S3).

Following the trial of influential study removal, there are some differences between the reported pooled effect estimates of the comparative meta-analyses. For ORs and AUCs alike, the differences between the PRS-only and mixed score studies appear to be minimized ( Supplementary Table S1). The improvement in the standardized ORs of the mixed scores is 0.04 from an initial 0.18, with the improvement in AUC estimation being 0.02 from 0.09. Although the subgroup and total heterogeneity were reduced for both analyses, the results need to be carefully interpreted, as between-study heterogeneity was still moderately high, and the removal of studies with high attributed heterogeneity also led to the removal of influential studies for the observed outcomes, including studies reporting outcomes from big datasets and using large SNP arrays (e.g., Galeotti et al. [19], Sharma et al. [6], Ke et al. [22]).

### 3.8. Addressing Publication Bias

To assess publication bias in the present study, we looked at the two meta-analyses (OR and AUC) separately. The contour-enhanced funnel plots for the two analyses can be seen in Supplementary Figure S3. The trim-and-fill method was utilized to correct for observed asymmetry. Egger’s regression test for funnel plot asymmetry indicates significant asymmetry for the OR meta-analysis (*p* = 0.0251). On the contrary, the AUC meta-analysis was not significantly affected by publication bias, as expressed by Egger’s test (*p* = 0.2906). Small-study bias appears significant in standardized OR analysis; however, the origin of bias must be carefully interpreted (see Section 4).

## 4. Discussion

### 4.1. Synthesis of Common-Variant Polygenic and Mixed Risk Scores for Pancreatic Cancer

This systematic literature review and meta-analysis evaluated the performance of risk prediction models for pancreatic cancer, synthesizing data from 23 PRS-only models and 10 mixed models that incorporated both genetic and clinical risk factors. Smoking, diabetes, BMI, age and sex were included in most models. The pooled OR for the PRS-only models was 1.40, which increased to 1.58 for the mixed models. While this suggests a potential enhancement in predictive accuracy with the addition of clinical factors, the difference in the ORs between the two groups was not significant (*p* = 0.15). In terms of discrimination, AUC values were reported in nine PRS-only models, with a pooled AUC of 0.61, and six mixed-models, with a pooled AUC of 0.70. This increase in the AUC was significant (*p* = 0.03), indicating that the combination of genetic and clinical data significantly improves their discriminative ability. This is a key finding due to the scarcity of studies comparing AUCs before and after inclusion of clinical risk factors. Of the studies included, only three reported an improvement in the AUC following the addition of clinical risk factors [7,20,26]. While all three studies recorded an improvement in the AUC following the addition of clinical risk factors, only Sharma et al. [7] formally assessed the significance of this improvement, which was in line with the findings of our synthesis.

The improvement in prediction metrics following the addition of clinical risk factors to PRSs has previously been observed in several phenotypes including cardiovascular disease, type 2 diabetes and breast cancer [57,58,59,60]. The non-significant difference in the ORs between the PRS-only and mixed models is most likely due to the high levels of heterogeneity seen in both the PRS-only (I^2^ = 95%) and mixed models (I^2^ = 88%), which may reflect varying study designs, population characteristics and the number of SNPs used. These factors impact the comparability of model performance and indicate that the standardization of PRS construction and the selection of clinical risk factors are needed. Clinical risk factor selection in mixed model studies is heterogenous, although we can see that age, sex, diabetes and smoking are common denominators in most mixed scores (Table 1). This is likely one of the sources of the between-study heterogeneity seen in all of the meta-analyses and subset analyses conducted. Future research efforts should focus on standardizing the selection of environmental risk factors for model building and emphasize uniformity in their measurement. Among the 27 studies included, 21 sourced their patient populations from large national or multinational biobanks, such as the UK Biobank, PanScan study, PANC I-III biobanks and the GERA cohort. The remaining studies were based on institutional or regional biobanks, which generally had smaller sample sizes and less population diversity that may introduce a higher risk of recruitment and population bias.

Authors have previously proposed the addition of more well-known clinical risk factors for pancreatic cancer, such as family history, alcohol consumption, history of chronic or acute pancreatitis and more [1,2,3]. Among these risk factors, there are metrics that remain underutilized in the prediction modeling of pancreatic cancer, including exercise status (relative to obesity and BMI), HbA1C levels (relative to diabetes and prediabetes diagnosis), patient history of pancreatitis, socioeconomic index and dietary habits. Disease cytopathology can be studied as an additional covariate, especially given the recent advancements in automated reporting and the creation of repositories [61]. Four studies included family history as a clinical risk factor [7,23,26,35], which has potential benefits for improving risk prediction but may also introduce some potential issues. Family history is a measure of inherited genetic risk, which may indicate that some genetic factors are missing from the PRS, but it could also result in the genetic information being double-counted and lead to inflated or inaccurate risk estimates. A well-designed mixed model would need to carefully separate out the genetic component of family history from the environmental factors such as shared lifestyle or exposures that also contribute to disease risk. Additionally, these risk factors can be used to perform multi-trait GWASs, which simultaneously analyze multiple correlated traits [62,63] such as BMI, diabetes and pancreatic cancer to refine estimated SNP effect sizes and/or detect novel SNPs. An ancestry subgroup analysis shows underrepresentation in non-Caucasian risk prediction modeling. Asian populations had lower prediction metrics (pooled AUC 0.62; 95% CI 0.57–0.67) than Caucasians (pooled AUC 0.72; 95% CI 0.60–0.84). Frameworks have been developed to improve model fit by including ancestry-differential effects in mixed populations [59,60]. A simulation study improved prostate cancer prediction by adding African-ancestry SNPs to those significant in Caucasian studies [61]. Despite these correction methods, non-Caucasians still have poorer metrics, possibly due to the use of Caucasian-derived SNPs, smaller sample sizes and data biases. Mixed ancestry populations are vital for validity, interpretability and clinical use [59,60,61]. The incorporation of clinical (environmental) risk factors in mixed model studies is characterized by a lack of uniformity in participant and risk factor selection, an observation that can account for the large estimated heterogeneity seen in our meta-analysis and can be considered a source of observation bias. It must be noted that the selection of risk factors is often dependent on the availability of such from each biobank studied. The uniformity of participant selection criteria such as age, measurement methods and definitions for risk factors (e.g., identification and definition of smokers) would greatly reduce between-study heterogeneity and enhance reproducibility in the future. Bias mitigation strategies must be adopted by all authors when constructing PRS studies. Bias assessment has been adequately addressed in most of the included studies since the research teams primarily work with anonymized data collected from large regions. Controlling for principal ancestry components is a common process in PRS development to adjust for the effect of between-participant relatedness. Random sampling, appropriate power analysis and sample size estimation, robust external validation processes and subgroup analyses are all key components that must be incorporated in future mixed risk score studies.

The number of SNPs used in polygenic risk scores (PRSs) varied widely across studies, ranging from 5 to 54 SNPs. Sharma et al. [6] and Zheng et al. [21] included the most SNPs in their PRS, with 49 and 54 SNPs, respectively. Although this variation did not significantly impact the effect sizes for pancreatic cancer risk, a greater number of SNPs was significantly positively associated with improved prediction performance (higher OR) in PRS-only models. This association was not observed in the mixed models, likely because the clinical risk factors, with larger effect sizes, overshadowed the genetic component. In mixed models, the PRS component was treated as a single variable, masking the impact of SNP count in meta-regression analysis. When analyzing AUC metrics, the number of SNPs in the PRS showed no significant association with the AUC, though larger PRS models suggested a positive trend. The lack of association may stem from the AUC’s focus on sensitivity and specificity rather than individual SNP effects.

Twin studies estimated that the heritability of pancreatic cancer was approximately 36% [63], with up to 10% of patients having an affected first-degree relative [64]. However, only 4.1% of the total phenotypic variation could be explained by the common variants identified to date by GWASs [62]. Recent findings suggest that rare variants with MAF < 0.01 contribute an additional 6.9% to heritability, while common variants account for 13.1% [62]. Predictive models could be enhanced by identifying these rare and common variants through well-powered GWASs and whole genome sequencing studies and incorporating them into PRS and mixed models [61,62].

A methodological study by Grund and Sabin [65] provided evidence that risk scores incorporating continuous variables often fail to appropriately transform these variables. As a result, the true effect of these variables may not be adequately captured in the final score. This may explain some of the discrepancies in model performance seen here. Standardizing ORs per unit increase in risk is crucial for comparing predictive performance across studies and subgroups and helps address the lack of variable transformation, which is one of the strengths of this meta-analysis.

Several authors have expanded on the misuse of AUCs and C-statistics in predictive risk scores [66,67,68], particularly in scores comprising many biomarkers with relatively small effect sizes. C-statistics and AUCs have proven to be insensitive to the addition of biomarkers with small effect sizes due to the calculation of the rank order of the predicted risks, as opposed to their effect size [67,68]. This observation is particularly true for common-variant PRSs comprising multiple weighed risk factors of small individual effect. Therefore, the observed significant improvement in the AUC prediction metrics for the mixed score studies can be attributed not only to the effect of clinical risk factors but also to the suppression of the individual effects of SNPs. A possible alternative to this approach is the inclusion of rare variants that tend to exhibit large individual associations.

Low sample sizes hinder the detection of large effect sizes from common SNP accumulation in polygenic scores. This is partly due to the difficulty in detecting rare variants linked with the phenotype and uncommon SNP combinations suggesting increased risk. A study by Aoki et al. [31] illustrates how ancestry, sample size and data regionality impact risk estimates. Smaller cohorts and limited regions may exaggerate SNP associations with pancreatic cancer. When its risks were converted to standardized ORs, their study was not an outlier, but it showed a wide confidence interval and small weight after synthesis.

### 4.2. Rare Variant Association in Pancreatic Cancer

The addition of rare variants within common-variant polygenic risk scores has also been shown to increase predictive metrics in different phenotypes [16,17]. Due to a lack of studies incorporating rare variants as part of genomic risk scores for pancreatic cancer, the present review is limited to individual rare-variant association studies [6]. Pathogenic and likely pathogenic germline variants were identified for the *APC*, *BRCA2*, *BUB1B*, *PALB2*, *STK11* and *ENG* genes [38,39,40,41,42,43,44,45,46,47,48,49,50,51]. Highly penetrant variants of the *APC* and *BRCA2* genes were also implicated in several rare variant association studies in pancreatic cancer patient families [29,30,31,32,33,34], indicating that they could be potential targets for new associations.

The combination of rare variations is an under-explored concept in risk prediction for pancreatic cancer. For instance, *ERCC4* variants in patients with *CDKN2A*-positive status [36] carry a higher burden. On the contrary, in *CDKN2A*-negative patients, the *ATM*, *BRCA2* and *PALB2* genes were found to have the highest number of germline pathogenic variants. The pathway-based aggregation of rare variants is predominant within the relevant studies, specifically for DNA repair pathways (*PALB2*, *BRCA1*, *ATM* genes) and tumor suppressor pathways (*CDKN2A*, *TP53* genes) [36,41,48,50]. Rare variant aggregation appears to be particularly important in familial pancreatic cancer, although their integration in risk prediction models remains a challenge.

Studies investigating rare variants in pancreatic cancer have focused on patient clusters from a single or few institutions, recording high effect sizes on the risk of pancreatic carcinoma. Most authors conclude that pancreatic carcinoma is highly heterogeneous. The identification of co-segregation in gene variants is another common finding between studies that warrants further investigation, as it seems that rare variations between genes may be linked, therefore multiplying the degree of predisposition to disease [9,47,69]. Several studies have suggested that the strength of association between rare variants and pancreatic cancer can even mean that they constitute potential future treatment targets or biomarkers for personalized medicine approaches in pancreatic cancer treatment [49,50,51,70]. Emerging themes and outstanding areas of improvement in rare variant association studies include the exponentially larger sample sizes required and the timing of sampling sequencing. The assessment of the functional impact of a rare variant is another hurdle towards clinical translation that is being resolved with in silico approaches of variable effectiveness [70]. The delineation of the functional interpretation of variants has been an important aspect of rare variant analysis in pancreatic cancer. Advances in multi-omic approaches including single-cell omics can help with the functional annotation of rare variants [13], and subsequently the construction of more robust polygenic scores [71].

Rare variant inclusion in polygenic risk scores is generally observed to result in a moderate improvement in the overall prediction metrics for phenotypes of interest, while significantly improving the classification of outlying individuals in the extremes of the selected phenotypes [72,73,74,75]. A recent study [72] looking into obesity in a European ancestry population achieved significant improvement in their prediction metrics of PRSs by adding rare variants to a common-variant PRS. Importantly, the rare-variant PRS was able to significantly increase the predictive capacity for individuals at risk of extreme obesity, confirming that rare variants are associated with phenotype outliers and can be useful in their risk stratification. Similarly, studies on lifetime risk of diabetes incorporating more than 20,000 SNPs in rare-variant PRSs exhibited significant improvement in the prediction of lifetime diabetes risk based on hemoglobin A1C [73,75]. The effect of rare variants in effectively delineating phenotypic outliers can be seen in the study by Smail et al. [76], the results of which indicate that rare-variant PRSs had an increased prediction risk of 20.8% for obesity and 62.3% for severe obesity when rare variants were added to a common-variant PRS. Risk stratification was also enhanced in cancer studies looking into breast and prostate cancer PRSs when rare variant analysis was incorporated [77,78]. The findings of these studies suggest that rare variants were found in participants within the low deciles of PRSs; however, they were so strongly associated with malignancy risk that further, selective symptomatic screening could be warranted despite the low overall PRS. Therefore, rare variation can potentially be utilized for pancreatic cancer risk prediction, possibly being able to identify individuals at risk for early-onset or biologically aggressive disease. Based on the results of robust studies on different malignancies, rare variant incorporation in PRSs has a distinct role to play in genetic risk stratification. Large-scale GWASs have indicated that rare variant screening can be valuable in common diseases in patients with low prior PRSs [69]. This observation might be more important in rare but deadly diseases, where a low common-variant PRS may lead to false reassurance, whereas the inclusion of rare variants can identify patient outliers with increased heritable disease burden and offer tailored surveillance strategies [38].

The continuous evolution of risk-based stratification is essential for tackling the challenges pancreatic cancer diagnosis poses. Rare variant association studies can potentially enhance prediction metrics and participant classification, but also advance individualized treatment strategies. Contemporary research on personalized treatment for pancreatic cancer has revealed that targeted molecular treatments have achieved comparable, and in some cases superior, survival outcomes compared to traditional chemotherapy regimens [79,80]. A recent pilot study on genomically selected targeted agent combinations [81] showed promising results, with most patients achieving a partial or complete response. In this context, polygenic risk scores that incorporate variants strongly associated with pancreatic cancer combined with functional information can potentially be expanded and utilized for the individualization of treatment strategies based on germline genetic data in addition to personalized screening. Therefore, the construction of complex mixed risk scores has the potential to constitute a source of information regarding treatment in addition to early detection.

## 5. Conclusions

Within this systematic review and meta-analysis, we obtained a thorough overview of the published studies looking at polygenic risk scores for pancreatic cancer risk prediction. Mixed scores that included clinical risk factors in addition to PRSs were shown to achieve superior discrimination metrics after a comparison of the pooled estimates. Standardization of the odds ratios revealed a potentially important increase in the prediction capacity for pancreatic cancer. The number of included SNPs in each polygenic score was significantly positively associated with the performance of the prediction score. An ancestry subgroup analysis revealed an underrepresentation of non-Caucasian populations in existing studies, with inferior prediction metrics when SNPs discovered in Caucasian populations were used for risk prediction in Asian populations. We have also presented several qualitative insights from rare variant association studies that indicate a potential for an improvement in prediction capacities in patient subpopulations. This is the first synthesis-based study to estimate the pooled effect of polygenic risk scores for the prediction of pancreatic cancer risk, as well as to identify score characteristics as potential contributors. Future research should focus on harmonizing methodologies and exploring the consistent, comprehensive integration of clinical variables to further enhance the predictive power and clinical applicability of PRSs for pancreatic cancer. This approach could enable the development of more robust, universally applicable models that offer meaningful risk stratification across diverse populations, paving the way for more personalized prevention and screening strategies.

## Figures and Tables

**Table 1:** Studies developing PRSs on pancreatic adenocarcinoma.

Author and Year	Dataset Origin	Sample Size	Clinical Risk Factors Investigated	SNPs	Summary Statistics
Yuan et al., 2022 [17]	Nurses’ Health Study HPFS Study PanScan/PanC4	5107 Cases 8845 Controls	Smoking, Obesity, T2DM (>2 years), Height, Blood Group, Exercise, Alcohol consumption No mixed score	22	<60 years OR: 6.91 >70 years OR: 4.12 Top vs. bottom centile
Sharma et al., 2022 [7]	UK Biobank	1042 Cases 10,420 Controls	Race, Smoking, DM, Family History, Waist Circumference Mixed score + subset analysis	49	HR: 2.738 Top vs. bottom quantile
Byrne et al. 2023 [5]	UK Biobank	451 Cases 195,822 Controls	Lifestyle Index (Weight, Activity, Dietary profile, Smoking status) Mixed Score	22	HR: 4.14 Top 5%
Fritsche et al., 2020 [18]	MGI Cohort UK Biobank Cohort	1265 Cases 446,955 Controls	No mixed score	10	OR: 1.64 (0.655–4.12) Top 1%
Nodari et al., 2023 [19]	PanScan I, II and III PANDoRA consortium	912 Cases 10,222 Controls	Smoking, Alcohol Consumption, T2DM No mixed score	6	<50 years OR: 2.90 (1.17–7.17) >50 years OR: 2.44 (1.76–3.38) 5th vs. 1st quintile
Galeotti et al., 2021 [20]	PanScan I,II,III and PanC4 dbGaP	2851 Cases 4810 Controls	Sex, Age, Smoking, T2DM Mixed Score	30	PRS OR: 3.24 (2.86–3.67) Mixed OR: 14.37 (5.57–37.09) 5th vs. 1st quantile
Pang et al., 2021 [21]	China Kadoorie Biobank	585 Cases 512,891 Controls	Age, Sex, Alcohol Consumption, Smoking, Education, BMI, Physical Activity, Diabetes (any) No mixed score	8	OR: 1.048 (1.05–1.11) per SD increase
Zeng et al., 2023 [22]	UK Biobank	1129 Cases 340,631 Controls	BMI, WC, Physical Activity, Sedentary Time, Dietary Habits, Alcohol Consumption, Smoking Separate clinical Score: Sex, Age, Education, Socioeconomic Status Mixed Score	54	HR: 1.58 (1.47–1.71) Intermediate vs. High PRS HR: 0.48 (0.41–0.56) Favorable vs. Unfavorable LS
Ke et al., 2023 [23]	UK Biobank 960 patients 257,348 controls (75% training, 25% testing)	1402 Cases 502,387 Controls	Gender, Age, Blood Group, Family history of bowel cancer, Smoking, Alcohol, BMI, Pancreatitis, T2DM, Gallbladder related disease, Hep B infection, Vitamin D deficiency, SLE Mixed Score	40	OR: 2.03 (1.65–2.50) 5th quintile vs. 1st quintile
Bogumil et al., 2020 [24]	Multiethnic Cohort Southern Community Cohort Study	691 Cases 13,778 Controls	None	11	OR: 2.25 (1.73–2.92) 5th vs. 1st quintile
Wang et al., 2020 [25]	Chinese, Single institution	254 Cases 1200 Controls	Age, Sex	21	OR: 1.36 (1.06–1.74)
Nakatochi et al., 2018 [26]	Japanese origin 945 patients, 2109 controls	945 Cases 2109 Controls	Smoking Family history of pancreatic cancer	5	OR: 1.98 (1.42–2.76) 1st vs. 5th quintile
Lu et al., 2021 [27]	PanScan I, II, III, PanC4 PANDoRA for replication	8769 Cases 7055 Controls	Sex, Age, Country	6	
Zhu et al., 2024 [28]	UK Biobank 765 cases	765 Cases 315,512 Controls	Smoking (stratification)		Non-smk OR: 2.31 (1.69–3.15) Ever-smk OR: 0.53 (0.30–0.91)
Pierce et al. 2023 [29]	PanScan I,II	1478 Cases 1534 Controls	None	12	OR: 0.9 (0.85–1.0)
Hu et al. 2024 [30]	UK Biobank	1402 Cases 409,532 Controls	Living status, Number in household, Dietary requirements, Basal metabolic rate, Overall health, Tense, Comparative, Hair color, Mean corpuscular hemoglobin, Vascular/heart problems, Reticulocyte count, Physical activity, Anxiety, Waist circumference, Cancer diagnosis, Lymphocyte count, Whole body fat free mass, Highest qualifications, Glycated hemoglobin	-	AUC: 0.549
Shi et al., 2019 [31]	The Cancer Genome Atlas Electronic Medical Records and Genomics	163 Cases 13,427 Controls	None	9	OR 1.67 (1.1–2.53) per SD increase
Aoki et al., 2021 [32]	Two centers in Brazil	78 Cases 256 Controls	Sex, Ethnicity, Smoking, Alcohol use, History of pancreatitis No mixed score	24	OR: 8.13 (2.95–22.43) 5th vs. 1st quintile
Graff et al., 2021 [33]	UK Biobank and GERA cohort	665 Cases 410,354 Controls	None	22	OR: 1.44 (1.33–1.55) per SD increase
Jia et al., 2020 [34]	UK Biobank	432 Cases 479,652 Controls	None	22	HR: 3.37 (2.39–4.76) 5th vs. 1st quntile
Kachuri et al., 2021 [35]	UK Biobank	768 Cases 413,753 Controls	Family History of Cancer, Smoking, Alcohol Intake, Adiposity	22	OR: 1.49 (1.37–1.63) per SD increase
Klein et al., 2018 [36]	PanC4	9040 Cases 12,496 Controls	None	22	OR: 2.20 (1.83–2.65) 5th vs. 10th decile
Meisner et al., 2020 [37]	UK Biobank	496 Cases 337,138 Controls	None	18	OR 1.42 (1.22–1.63) per SD increase
Rothwell et al., 2022 [38]	UK Biobank	478 Cases 366,016 Controls	Metabolic Syndrome, Height, Alcohol, Smoking, Education Levels Not used for mixed modeling	26	Used only for stratifying. No separate report
Salvatore et al., 2021 [39]	MGI UK Biobank	1089 Cases 430,570 Controls	BMI, Alcohol, Smoking Mixed Score	18	OR: 2.16 (1.76–2.65) 5th vs. 1st quintile
Wang et al., 2022 [40]	Penn Medicine Biobank	46 Cases 6383 Controls	Stratified for age and sex	18	OR: 1.023 (0.759–1.377) per SD increase
Dite et al., 2023 [41]	UK Biobank	851 Cases 376,462 Controls	None	22	OR: 1.31 (95% CI, 1.264–1.357) per SD increase

**Table 2:** Studies on rare variant associations with pancreatic adenocarcinoma.

Author and Year	Genes and Variants Investigated	
Saha et al., 2020 [42]	*KMT2C, TP53*, NM_000546.6(*TP53*):c.413C>T, *SMAD4*, *CTNNB1, ERBB2, NOTCH1, PARP1, PIK3R1, PIK3CD*	
Symonds et al., 2022 [43]	*ERCC4, RET, HMBS, EPCAM, MRE11, ATM* (rs1555093684), *BRCA2* (rs80359537), *CDKN2A*, *CDC73, ERCC4, ERCC5, FANCD2*, NM_001018115.3(*FANCD2*):c.1876C>T, *L2HGDH*, *PMS2*, NM_000535.7(*PMS2*):c.904-2A>G, NM_000535.7(*PMS2*):c.1882C>T, *RB1*, *CFTR* (rs201958172), *GBA* (rs367968666), *HMBS*, *MRE11, NSD1, PALB2*, NM_024675.4(*PALB2*):c.470C>A, *RAD51C, RET*, *SLC25A13*, NM_014251.3(*SLC25A13*):c.1762C>T (p.Arg588Ter), NM_014251.3(*SLC25A13*):c.1063C>T (p.Arg355Ter), *SLX4*, *TRIM37*, (p.Ser157Ter), NM_000283.4(*PDE6B*):c.2395C>T (p.Arg799Ter), NM_020975.4(*RET*):c.[2410G>A;2832C>G], NM_015047.3(*EMC1*):c.2059C>T (p.Arg687Ter)	
Gentiluomo et al., 2019 [11]	*TAS1R2* (rs11261087)	
Tan et al., 2022 [12]	*ATM, POLE, BRCA2, TYR03, PABC1, SSC5D*	
Slater et al., 2021 [9]	*PCMS* c.724G>A, FPC p.(*A242T*)	
Yang et al. 2016 [44]	*APC, ATM* (rs587779852, rs567060474), *BRCA1* (rs28897673), *BRCA2* (rs80359537), *CASR*, *CFTR* (rs201958172, rs397508725, rs151048781, rs149279509), *CPA1, FANCA, MLH1, MSH2* (rs267607911, rs145649774, rs34136999), *MSH6* (rs376220212), *PALB2* (rs180177100), *PMS2* (rs587780046), *TP53* (rs145151284)	
Grant et al., 2018 [45]	*BRCA2* (rs80359550), *PZP, IFNA5, FGFR3* (rs17881656), NM_000059.4 (*FGFR3*):c.10095	
Obazee et al., 2019 [46]	*BRCA2* (rs11571833), *CHEK2* (rs17879961)	
Wieme et al. 2021 [47]	*ATM*, NM_000051.4(*ATM*):c.1564_1565del(p.Glu522fs),NM_000051.4:c.3756_3757dup,NM_000051.4(*ATM*):c.6095G>A(p.Arg2032Lys),NM_000051.4:c.6096-9_6096-5del, NM_000051.4(*ATM*):c.7327C>T(p.Arg2443Ter), NM_000051.4(*ATM*):c.8494C>Tp.Arg2832Cys), *BRCA1*, NM_007294.4(*BRCA1*):c.2411_2412del(p.Gln804fs), NM_007294.4(*BRCA1*):c.3991C>T(p.Gln1331Ter), NM_007294.4(*BRCA1*):c.3770_3771del(p.Glu1257fs), *BRCA2*, NM_000059.4(*BRCA2*):c.8487+47C>Tc.1989del,NM_000059.4(*BRCA2*):c.3545_3546del(p.Gln1181_Phe1182insTer), NM_000059.4(*BRCA2*):c.475G>A(p.Val159Met), NM_000059.4(*BRCA2*):c.4935del(p.Glu1646fs), NM_000059.4(*BRCA2*):c.5073dup(p.Trp1692fs), NM_000059.4(*BRCA2*):c.6275_6276del(p.Leu2092fs), NM_000059.4:c.7415dup, NM_000059.4(*BRCA2*):c.7480C>T(p.Arg2494Ter), NM_000059.4(*BRCA2*):c.8167G>C(p.Asp2723His)c.8755-1G>A, NM_000059.4(BRCA2):c.8755-1G>T, CHEK2, NM_007194.4:c.846+4_846+7del, NM_007194.4(*CHEK2*):c.917G>C(p.Gly306Ala), *ERCC4, FANCE, NBN*, NM_031483.7(*ITCH*):c.212+3A>Gc.2263G>T, NM_000535.7(*PMS2*):c.137G>T(p.Ser46Ile), NM_000535.7(*PMS2*):c.1840A>T(p.Lys614Ter), NM_000546.6(*TP53*):c.528C>A(p.Cys176Ter), NM_000488.4(*SERPINC1*):c.801_805del(p.Lys268fs)	
Yu et al. 2022 [48]	*ADGRF3* (rs150568284), *MAP3K11* (rs35487354), *TERT* (rs2736098), rs9579139, *PRHOXNB*, *PDX1* (rs9581943), *HECTD2* (rs61754654), *INPP4A*, *ABCB1*, *TAAR6* (rs41298393), *SBDS* (rs120074160), *OR2AT4* (rs145245435), *GPR113*, chr7-87160645-87160645	
Hendifar et al. 2021 [49]	*V600E*, *BRAF*, *SND1*-*BRAF* fusions	
Puccini et al., 2022 [50]	*ATM*, *BRCA1*, *BRCA2*, *CDKN2A*, *FANCA*, *AD50*	
Tan et al., 2021 [12]	*PALD1*, *COL4A2* (rs370540848)—*CYLC2* (rs139628328), *ZFYVE9* (rs749151241)—*BRD3* (rs149702159)—*BRD4* (rs149982995, rs35676845), *LRP1B* (rs148734150, rs139901295, rs75995642, rs77234491, rs138996626)	
Horn et al., 2021 [51]	*CDK9A* nsv6313094	
Hei Ryu et al., 2021 [52]	*ATM* (rs587781598, rs780619951, rs1442299125, rs786202800, rs770641163), BRCA1 (rs80357662, rs80357524), *BRCA2* (rs80359659, rs80358427), *MSH3* (rs539295465), *RAD50* (rs778555849), *RAD51D* (rs1403784434, rs753862052), *PALB2* (rs587776527), *PMS2* (rs587778618), *SPINK1* (rs148954387), *TP53* (rs397516436)	
Fujitani et al., 2023 [10]	*APC*, *BRCA2* (rs2072490081), *BUB1B* (rs28989182), *MSH6* (rs63750909), *AXIN2*, *BMPR1A*, *MSH2* (rs772662439), *SMARCA4*, *TP53* (rs397516436)	
McWilliams et al., 2018 [53]	*CDKN2A*	
Kawamoto et al., 2021 [54]	*CPA1* (rs267601284, rs782248213, rs184981267, rs201750163), *CPB1* (rs1275676207, rs201519774)	
Chen et al., 2019 [55]	*TERT*-*CPLTML* (rs35226131), DAB2 (rs2255280)	
Polaski et al., 2021 [56]	*TERT* (rs377639087)	
Yu et al., 2021 [8]	*HECTD2* (rs61754654), *CSGALANACT1*_DUP_01 (rs34799877), *PFAS* (rs34778863), *PLEKHH1* (rs45616031), *INPP4A*, *TMEM217* (rs34322528), *OR2AT4* (rs145245435), *PDGFB* (rs55634318), *GPR113* (rs150568284), *SPTBN5* (rs149954264), *UBE3B* (rs61748069)	

## Supplementary Materials

The following supporting information can be downloaded at https://www.mdpi.com/article/10.3390/cancers17020241/s1, Table S1: Search strategies for studies on common-variant and mixed PRSs in pancreatic cancer; Table S2: Search strategies for studies on rare variant associations in pancreatic adenocarcinoma; Table S3: Comparison of pooled study estimates before and after influential heterogenous study removal for OR and AUC reports; Figure S1: Comparison of pooled AUC metrics between Caucasian and Asian ancestry PRS-only models; Figure S2: Comparison of pooled AUC metrics between Caucasian and Asian ancestry mixed models; Figure S3: Funnel plots following trim and fill process for OR meta-analysis (Egger’s test intercept = 3.67) (A), and AUC meta-analysis (Egger’s test intercept = -6.467) (B).
