# Supplementary material for: Utility of Polygenic Risk Scores (PRSs) in Predicting Pancreatic Cancer: A Systematic Review and Meta-Analysis of Common-Variant and Mixed Scores with Insights into Rare Variant Analysis
Source: Cancers (Basel). 2025 Jan 13;17(2):241. doi: 10.3390/cancers17020241 (PMC11764467; doi:10.3390/cancers17020241)
Supplement: Supplementary file 1 [file cancers-17-00241-s001.zip › cancers-3366084-supplementary.pdf]

Supplemental Table S1: Search strategies for studies on common variant and mixed PRS in pancreatic cancer

MEDLINE
(polygenic risk score) OR (PRS) OR (PGS) OR (polygenic score) OR (SNP score)) AND ((pancreatic cancer) OR (pancreatic carcinoma) OR (pancreatic adenocarcinoma))
**Ovid**
((polygenic risk score or PRS or PGS or polygenic score or SNP score) and (pancreatic cancer or pancreatic carcinoma or pancreatic adenocarcinoma)).mp. [mp=ti, ab, tx, ct, sh, hw, tn, ot, dm, mf, dv, kf, fx, dq, bt, nm, ox, px, rx, an, ui, sy, ux, mx]
**Web of Science**
(((((ALL=(pancreatic cancer)) OR ALL=(pancreatic adenocarcinoma)) OR ALL=(pancreatic mass)) AND ALL=(polygenic risk score)) OR ALL=(PRS)) OR ALL=(genetic risk score))))
**Embase**
ALL (pancraetic AND cancer) OR ALL (pancreatic AND carcinoma) OR ALL (pancreatic AND adenocarcinoma) AND ALL (polygenic AND risk AND score) OR ALL (prs)

Supplemental Table S2: Search strategies for studies on rare variant associations in pancreatic adenocarcinoma

**MEDLINE**
((pancreatic adenocarcinoma) OR (pancreatic cancer) OR (pancreatic ductal adenocarcinoma)) AND ((rare variants) OR (rare variants analysis) OR (rare genetic variation))
**Ovid**
((polygenic risk score or rare variants or PGS or polygenic score or rare SNP) and (pancreatic cancer or pancreatic carcinoma or pancreatic adenocarcinoma)).mp. [mp=ti, ab, tx, ct, sh, hw, tn, ot, dm, mf, dv, kf, fx, dq, bt, nm, ox, px, rx, an, ui, sy, ux, mx]
**Web of Science**
(((((ALL=(pancreatic cancer)) OR ALL=(pancreatic adenocarcinoma)) OR ALL=(pancreatic mass)) AND ALL=(rare variants)) OR ALL=(PRS)) OR ALL=(rare variants analysis))))
**Embase**
(ALL (pancreatic AND cancer) OR ALL (pancreatic AND adenocarcinoma) OR ALL (pancreatic AND ductal AND adenocarcinoma)) AND (ALL (rare AND variants AND analysis) OR ALL (rare AND genetic AND variants) OR ALL (rare AND snp)) AND PUBYEAR > 2013 AND PUBYEAR < 2025 AND (LIMIT-TO (SUBJAREA , "BIOC") OR LIMIT-TO (SUBJAREA , "MEDI")) AND (LIMIT-TO (DOCTYPE , "ar")) AND (LIMIT-TO (EXACTKEYWORD , "Human")) AND (LIMIT-TO (LANGUAGE , "English"))

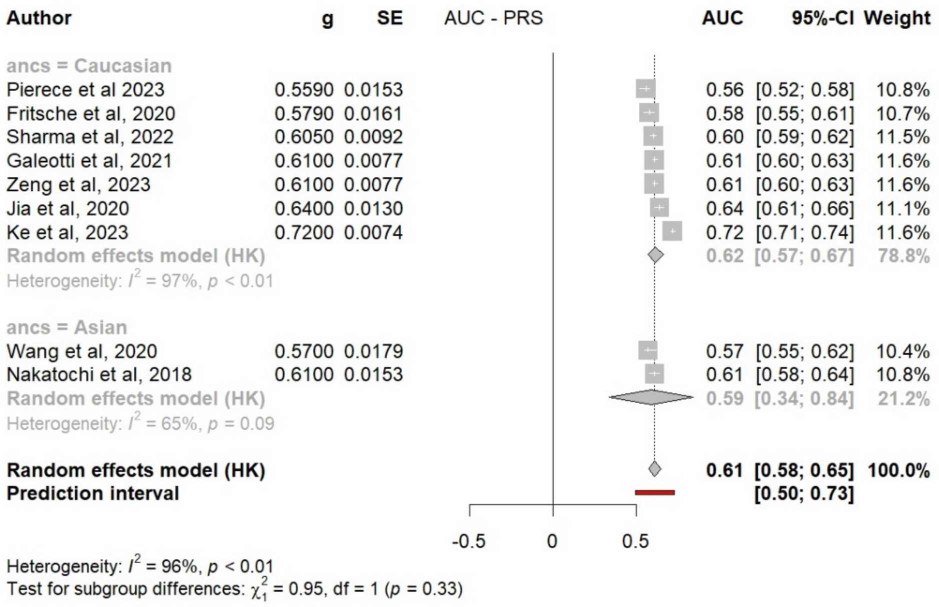

Supplemental Figure S1: Comparison of pooled AUC metrics between Caucasian and Asian ancestry PRS only models

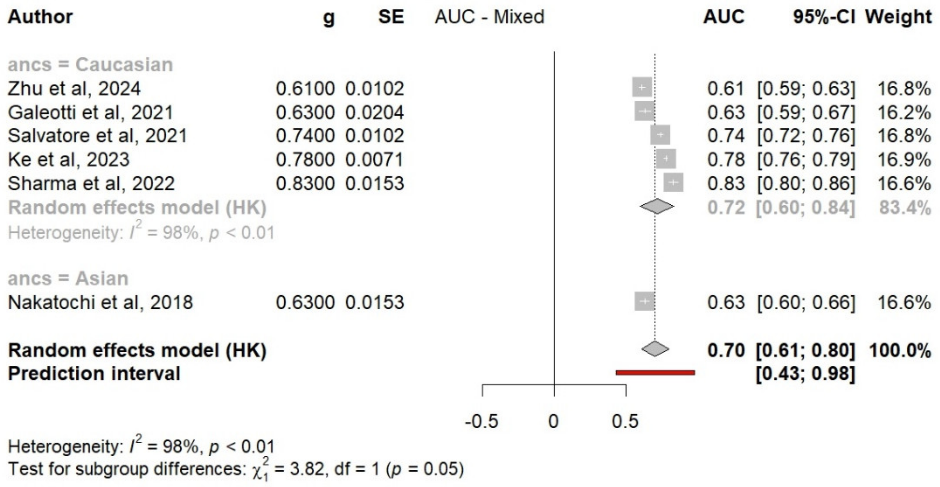

Supplemental Figure S2: Comparison of pooled AUC metrics between Caucasian and Asian ancestry mixed models

Supplemental Table S3: Comparison of pooled study estimates before and after influential heterogenous study removal for OR and AUC reports

Analysis	Effect Size	95%CI	*p*-value	I^2
**Main Analysis OR**	1.45	1.34-1.57	0.15	95%
PRS-Only Studies	1.40	1.28-1.53		71.1%
Mixed Score Studies	1.58	1.34-1.88		28.9%
**Study Outliers Removed**	1.45	1.39-1.51	0.49	71.2%
PRS-Only Studies	1.44	1.37-1.51		58.9%
Mixed Score Studies	1.48	1.35-1.63		83.7%
**Main Analysis AUC**	0.65	0.60-0.69	0.03	98%
PRS-Only Studies	0.61	0.58-0.65		96%
Mixed Score Studies	0.70	0.61-0.80		98%
**Study Outliers Removed**	0.61	0.59-0.62	0.02	47.1%
PRS-Only Studies	0.60	0.58-0.62		57.4%
Mixed Score Studies	0.62	0.58-0.64		0%

Effect size and 95%CIs correspond to total pooled estimates and grouped pooled estimates in the corresponding rows. P-values: from chi-squared test for pairwise subgroup differences. I^2: Higgin's test for between-study heterogeneity.

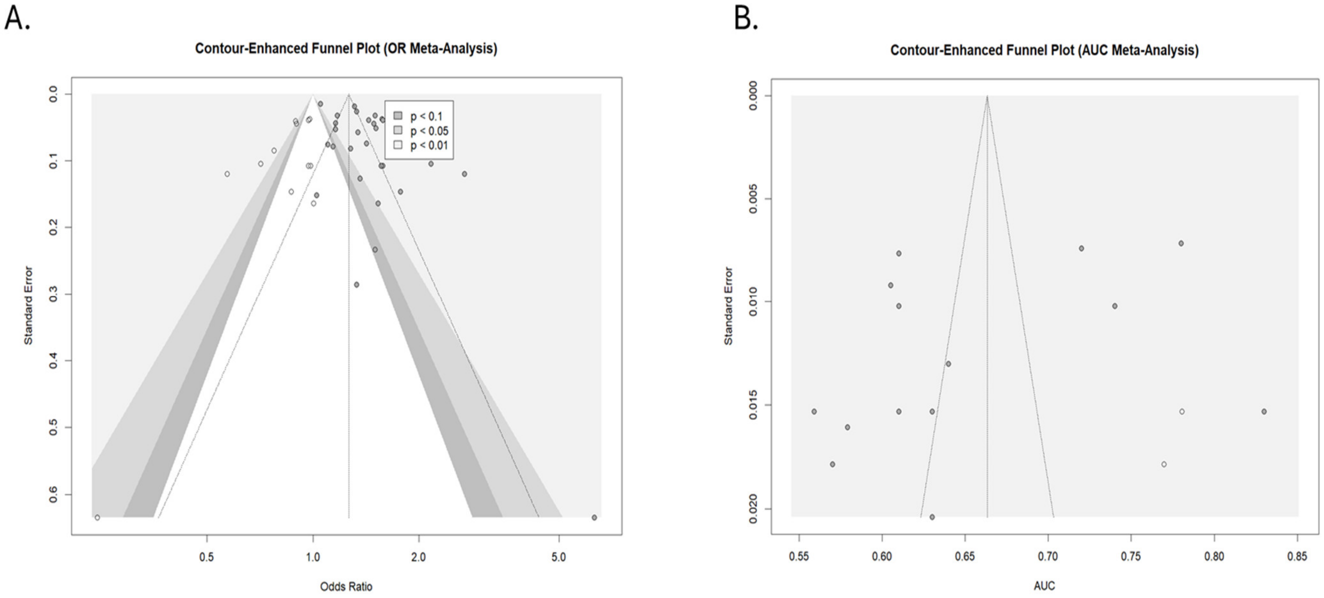

Supplemental Figure S3: Funnel plots following trim and fill process for OR meta-analysis (Egger's test intercept = 3.67) (A), and AUC meta-analysis (Egger's test intercept = -6.467) (B)
